# The Effect of Fluorides (BaF_2_, MgF_2_, AlF_3_) on Structural and Luminescent Properties of Er^3+^-Doped Gallo-Germanate Glass

**DOI:** 10.3390/ma15155230

**Published:** 2022-07-28

**Authors:** Magdalena Leśniak, Gabriela Mach, Bartłomiej Starzyk, Karolina Sadowska, Tomasz Ragiń, Jacek Żmojda, Marcin Kochanowicz, Marta Kuwik, Piotr Miluski, Gloria Lesly Jimenez, Agata Baranowska, Jan Dorosz, Wojciech Pisarski, Joanna Pisarska, Zbigniew Olejniczak, Dominik Dorosz

**Affiliations:** 1 Faculty of Materials Science and Ceramics, AGH University of Science and Technology, 30 Mickiewicza Av., 30-059 Krakow, Poland; machgabriela1@gmail.com (G.M.); starzyk@agh.edu.pl (B.S.); glesly@agh.edu.pl (G.L.J.); ddorosz@agh.edu.pl (D.D.); 2 Faculty of Electrical Engineering, Bialystok University of Technology, 45D Wiejska Street, 15-351 Bialystok, Poland; k.sadowska@doktoranci.pb.edu.pl (K.S.); tomasz.ragin@pb.edu.pl (T.R.); j.zmojda@pb.edu.pl (J.Ż.); m.kochanowicz@pb.edu.pl (M.K.); p.miluski@pb.edu.pl (P.M.); doroszjan@pb.edu.pl (J.D.); 3 Institute of Chemistry, University of Silesia, 9 Szkolna Street, 40-007 Katowice, Poland; marta.kuwik@ud.edu.pl (M.K.); wojciech.pisarski@us.edu.pl (W.P.); joanna.pisarska@us.edu.pl (J.P.); 4 Faculty of Mechanical Engineering, Bialystok University of Technology, 45C Wiejska Street, 15-351 Bialystok, Poland; a.baranowska@pb.edu.pl; 5 Institute of Nuclear Physics, Polish Academy of Sciences, Radzikowskiego 152, 31-342 Krakow, Poland; zbigniew.olejniczak@ifj.edu.pl

**Keywords:** heavy metal oxide glasses, gallo-germanate glass, BaF_2_, MgF_2_, AlF_3_, DSC, thermal stability, structure, IR, Raman, ^27^Al MAS NMR spectrum, luminescence properties, erbium ions, wide emission

## Abstract

The effect of BaF_2_, MgF_2_, and AlF_3_ on the structural and luminescent properties of gallo-germanate glass (BGG) doped with erbium ions was investigated. A detailed analysis of infrared and Raman spectra shows that the local environment of erbium ions in the glass was influenced mainly by [GeO]_4_ and [GeO]_6_ units. Moreover, the highest number of non-bridging oxygens was found in the network of the BGG glass modified by MgF_2_. The ^27^Al MAS NMR spectrum of BGG glass with AlF_3_ suggests the presence of aluminum in tetra-, penta-, and octahedral coordination geometry. Therefore, the probability of the ^4^I_13/2_→^4^I_15/2_ transition of Er^3+^ ions increases in the BGG + MgF_2_ glass system. On the other hand, the luminescence spectra showed that the fluoride modifiers lead to an enhancement in the emission of each analyzed transition when different excitation sources are employed (808 nm and 980 nm). The analysis of energy transfer mechanisms shows that the fluoride compounds promote the emission intensity in different channels. These results represent a strong base for designing glasses with unique luminescent properties.

## 1. Introduction

One of the most popular techniques for designing glass for photonic applications is the modification of their structural properties. This is more significant in luminescent materials because the quantum efficiency of active dopants has a strong dependence on the chemical bonding type. In the past, several studies on oxide gallo-germanate glasses have been reported due to their potential use in photonics. The properties of this kind of glass have been explored in Ga_2_O_3_–GeO_2_–BaO [1], Ga_2_O_3_–GeO_2_–BaO–K_2_O [2], Ga_2_O_3_–GeO_2_–La_2_O_3_–BaO [3,4], and Ga_2_O_3_–GeO_2_–BaO–La_2_O_3_–Y_2_O_3_ [5] systems, demonstrating that gallo-germanate glasses offer wide optical transparency (∼6 µm), low phonon energy (~850 cm^−1^), and a high linear and nonlinear refractive index [6]. Moreover, gallo-germanate glasses are mechanically resistant and chemically stable, which allows their use in the production of fibers and waveguides [7,8,9].

It is well known that the presence of fluoride compounds in oxy-fluoride gallo-germanate glasses doped with rare earth ions (RE ions) affects the spectroscopic properties of the glass, due to the chemical affinity between the dopants (RE ions) and ligands (fluorides). However, their presence in the oxide glass network reduces the phonon energy, decreases non-radiative decay, and increases the emission intensity, triggering higher quantum efficiencies. On the other hand, their effect on the luminescent properties of gallo-germanate glass (BGG) doped with RE ions has been focused on the use of barium difluoride (BaF_2_); it has been found that its presence can modify the glass composition, which impacts the efficiency of the emission, optical transmission, and spectra profile [10,11,12,13].

Among the lanthanides, erbium is the most intensively studied element in luminescence because of its wide range of applications as an optical fiber amplifier, NIR fiber laser [14,15], optical temperature sensor [16], non-linear optical device [17], and up-conversion luminescent source [18] due to its unique properties. One of the most attractive applications is its use as an EDFA amplifier working in the III telecommunication window, due to its emission at 1550 nm originating from the ^4^I_13/2_ → ^4^I_15/2_ transition; however, its low solubility and the high-phonon energy of silicon glasses as a matrix limit the optical gain and spectral width of the amplification band [19]. For this reason, the interest in the development of new soft glasses that are able to accept a higher erbium concentration, characterized by a broadband emission in the near-infrared range (NIR), has grown [20,21], and it has been found that the use of gallo-germanate glass with BaF_2_ showed an increase in the linewidth at 1.55 µm from 50 nm to 67 nm [22].

Additionally, its ability to convert near-infrared light into green and red light as a result of the up-conversion (UC) processes increases its interest [23]. In this case, if the population of higher energy states mainly occurs by the excited state absorption (ESA) and the energy transfer with up-conversion (ETU), then the green and red emission bands will be a trigger. The quantum efficiency of these transitions strictly depends on the structural properties of the host and is higher when the phonon energy is lower [24,25].

In this work, we focus on the role of different fluoride compounds (BaF_2_, MgF_2_ AlF_3_) in the structural properties of gallo-germanate glass and analyzed for a possible correlation of these features with the intensity of radiative transitions in erbium ions. For a better understanding of the energy transfer mechanism, we used the two main excitation schemes of Er^3+^ ions (800 and 980 nm), which corresponds to the ^4^I_15/2_ → ^4^I_9/2_ and ^4^I_15/2_ → ^4^I_11/2_ transitions, respectively. Moreover, we have investigated the structure-luminescence properties of Er^3+^-doped glasses as a function of the BaO/MF ratio, where MF = AlF_3_, BaF_2_, MgF_2_, whose cation sizes are: 1.44 Å (Ba^2+^); 0.66 Å (Mg^2+^) and 0.47 Å (Al^3+^/IV); and 0.56 Å (Al^3+^/V), and 0.61 Å (Al^3+^/VI).

## 2. Materials and Methods

Gallo-germanate glasses doped with Er_2_O_3_ and modified by BaF_3_, MgF_2_, and AlF_3_ were synthesized using the conventional melt-quenching. The chemical composition of the glasses is as follows: (29.5-x) BaO—60 Ge_2_O_3_—10 Ga_2_O_3_—x BaF_2_/MgF_2_/AlF_3_—0.5 Er_2_O_3_, where x = 0 or 10 (in mol%). The glasses were labeled according to their chemical compositions. The first letters of the BaO, Ga_2_O_3_, and GeO_2_ oxides, the molar content of erbium, and the corresponding fluoride were used to name the samples. In this paper, the following names of the samples were used: BGG0.5Er glass (gallo-germanate glass doped with 0.5 mol% of Er_2_O_3_), BGG0.5Er_BaF_2_ glass (gallo-germanate glass doped with 0.5 mol% of Er_2_O_3_ and modified by BaF_2_), BGG0.5Er_MgF_2_ glass (gallo-germanate glass doped with 0.5 mol% of Er_2_O_3_ and modified by MgF_2_), and BGG0.5Er_AlF_3_ glass (gallo-germanate glass doped with Er_2_O_3_ and modified by 0.5 mol% of AlF_3_). Each set of 6 g was prepared employing materials from SigmaAldrich (Saint Louis, MI, USA) with a purity of >99.99% (BaO, Ge_2_O_3_, Ga_2_O_3_, BaF_2_, MgF_2_, AlF_3_, Er_2_O_3_). The components were homogenized in an agate mortar and then melted in a platinum crucible at 1250 °C for 1.5 h in an electric furnace. Finally, the melt was poured into a brass mold preheated at Tg-30 °C and annealed for 24 h (at Tg-30 °C) to remove residual internal stress.

The X-ray diffraction patterns were obtained using an X’Pert Pro X-ray diffractometer supplied by PANalytical (Almelo, The Netherlands) with Cu Kα1 radiation (λ = 1.54056 Å) in the 2θ range of 10–90°. The X-ray tube was operated at 40 kV and 40 mA and equipped with a scintillation detector (Almelo, The Netherlands) to measure the intensity of the scattered X-rays.

The differential scanning calorimetry (DSC) curves were obtained in the range of 200–1050 °C at 10 °C/min using the SETARAM Labsys thermal analyzer (Setaram Instrumentation, Caluire, France). Measurements were carried out with an uncertainty of ±1 °C. The glass transition Tg (onset) and the crystallization Tx temperatures were estimated at the onset and maximum of peaks, respectively. Based on these temperatures, the stability factor ∆T was calculated.

The infrared (IR) spectra of the glasses were measured with the Fourier spectrometer (Bruker Optics-Vertex70V, Rheinstetten, Germany) using the KBr pellet technique. All spectra were recorded at 128 scans with a resolution of 4 cm^−1^. The Raman spectra of the glasses were obtained using a LabRAM HR spectrometer (HORIBA Jobin Yvon, Palaiseau, France) with an excitation wavelength of 532 nm. The diffraction grating was 1800 lines/mm. The Raman spectra were recorded with the standard spot of about 1 µm. The IR and Raman’s spectra were normalized and deconvoluted using Fityk software (0.9.8, open-source (GPL2+)).

The luminescence spectra were measured using the JobinYvon Fluoromax4 spectrophotometer (Horiba Jobin Yvon, Longjumeau, France).

## 3. Results

### 3.1. X-ray Diffraction

**Figure 1:**
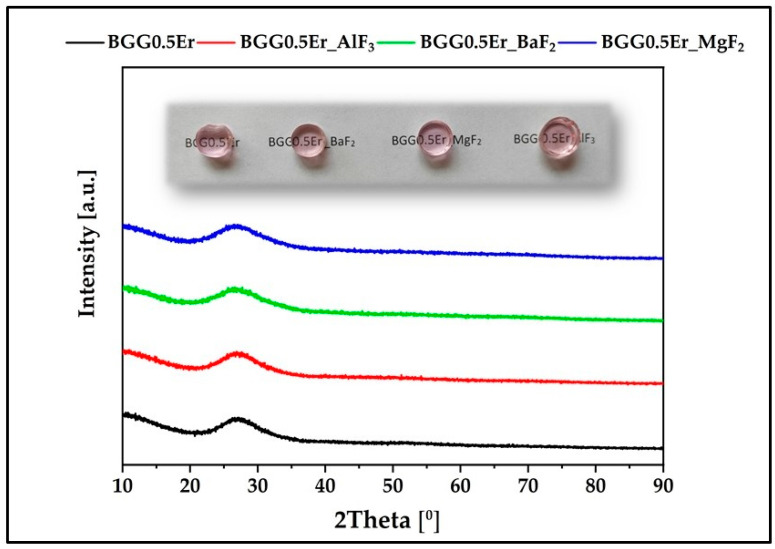
X-ray diffraction patterns of glasses. Pictures of the fabricated glasses (inset).

The X-ray diffraction patterns of the erbium-doped glasses are shown in Figure 1. Each pattern confirmed the absence of the crystalline phase because only broad humps around 28° are observed, which corroborates the amorphous character of all the samples [26]. Moreover, pictures of the fabricated glasses are presented in the inset of Figure 1.

### 3.2. DSC

**Figure 2:**
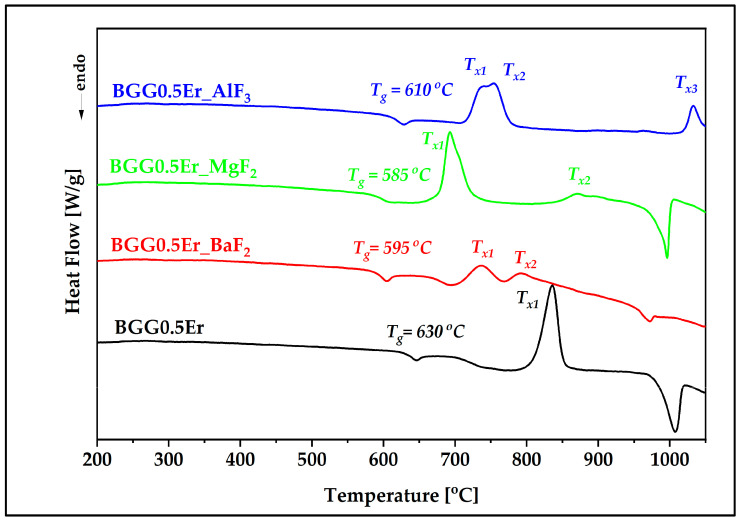
DSC curves of BGG0.5Er glass modified by BaF_2_, MgF_2_, and AlF_3_.

Differential scanning calorimetry (DSC) curves in the range of 200–1050 °C are presented in Figure 2. The DSC curves show that the exothermic behavior changes as a function of the fluoride component employed. In the case of the BGG0.5Er oxide glass, just one exothermic peak (T_x_) was observed at 836 °C while the BGG0.5Er_BaF_2_ (at 735 °C, 792 °C) and BGG0.5Er_MgF_2_ (at 642 °C, 871 °C) glasses exhibited two; and BGG0.5Er_AlF_3_ displayed three T_x_ (at 735 °C, 754 °C, and 1030 °C). These could be caused by the valence of the cations Ba^2+^, Mg^2+^, and Al^3+^ within the matrices.

The thermal stability (ΔT) of a glass can be determined as the difference between T_x1_ and T_g_; the higher the ΔT parameter, the greater the probability of successful use of the material to produce optical fibers [27,28]. As expected, the BGG0.5Er glass has the highest thermal stability (ΔT = 206 °C), which decreases with the presence of monovalent fluorine anions up to 57 °C (BGG0.5Er_MgF_2_ glass) [29]. The T_g_ and T_x_ temperatures of all the fluorine anions related to the first endothermic and exothermic peaks, as well as thermal stability ΔT of the glasses, are presented in Table 1.

### 3.3. Structural Analysis

Previous reports showed that the network of the BGG glass forms a ring structure that is composed of connected GeO_4_ and GaO_4_ tetrahedra and surrounded by alkaline earth (Ba^2+^) or rare earth ions that act as charge compensators for the negative charge of the gallate tetrahedra. Depending on the M/Ga cation ratio (where M is alkali metal or alkaline earth metals), gallium cations tend to balance the charges, forming four- or six-fold coordination units. Moreover, the Raman spectra of the gallo-germanate glasses show that four different structures describe the network of this glass (Q^n^, where n = 0, 1, 2, and 3, which corresponds to the number of bridging oxygen) [2,5,13,30,31,32]. This study discusses the glass network related to the IR and Raman spectra of glasses based on the network model mentioned above.

#### 3.3.1. IR Spectra

**Figure 3:**
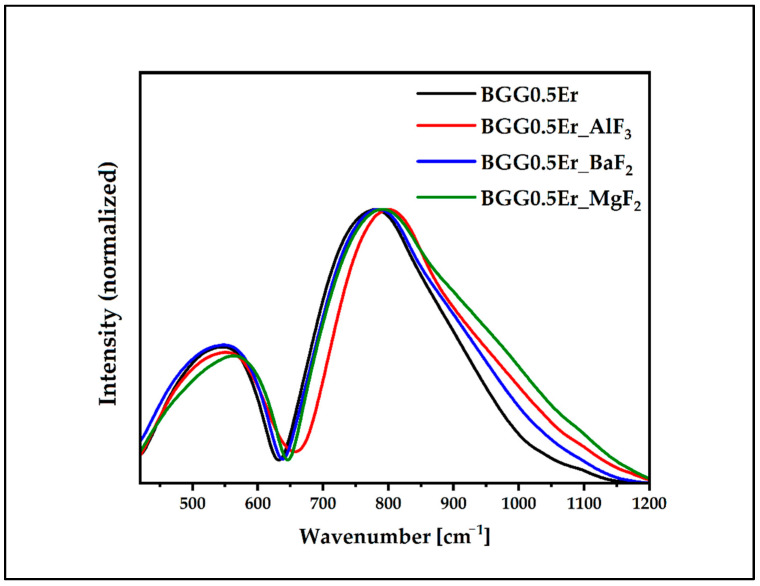
Normalized IR spectra of glasses in the 420 cm^−1^–1200 cm^−1^ range.

The normalized IR spectra in the 420 cm^−1^–1200 cm^−1^ range of the BGG0.5Er glass with and without the BaF_2_, AlF_3_, and MgF_2_ compounds are shown in Figure 3. All of them exhibited two domains (bands) at the (1) 420 cm^−1^–600 cm^−1^ and (2) 650 cm^−1^–950 cm^−1^ regions. These do not overlap, which means that the structural modification occurred after adding the BaF_2_, MgF_2_, and AlF_3_ compounds to the BGG0.5Er glass matrix.

**Figure 4:**
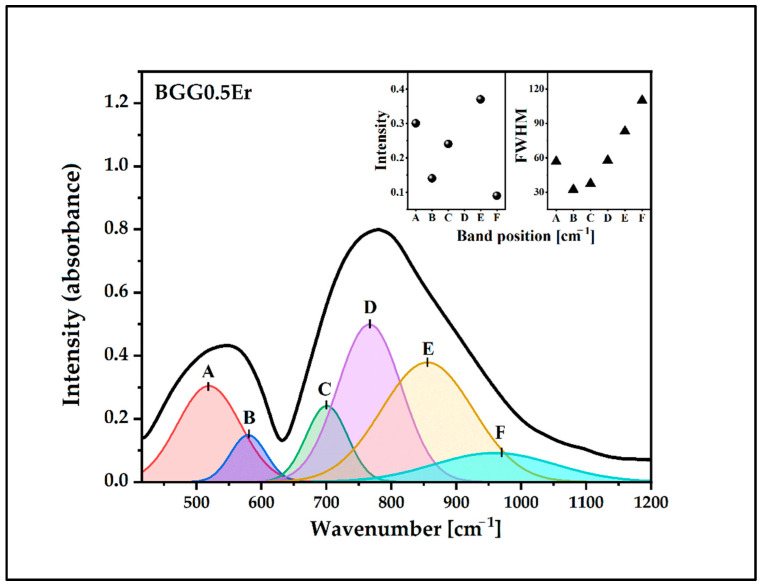
Deconvoluted IR spectrum of BGG0.5Er glass.

**Figure 5:**
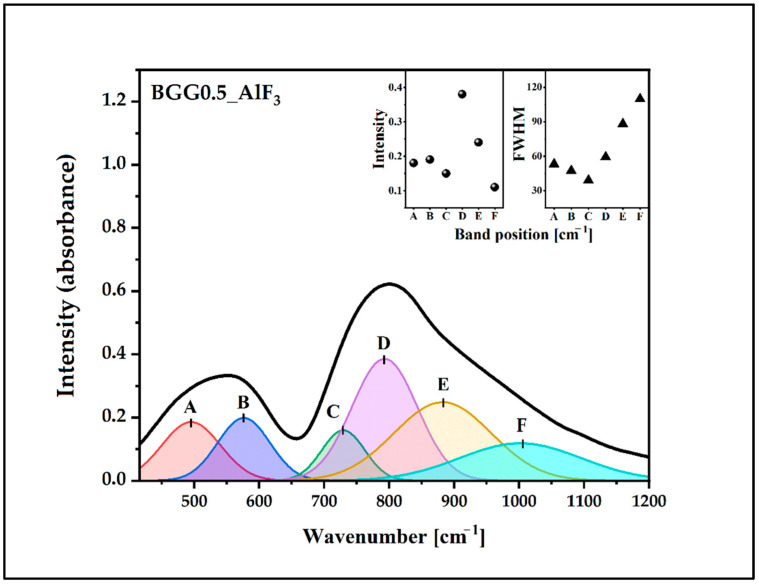
Deconvoluted IR spectrum of BGG0.5Er_AlF_3_ glass.

**Figure 6:**
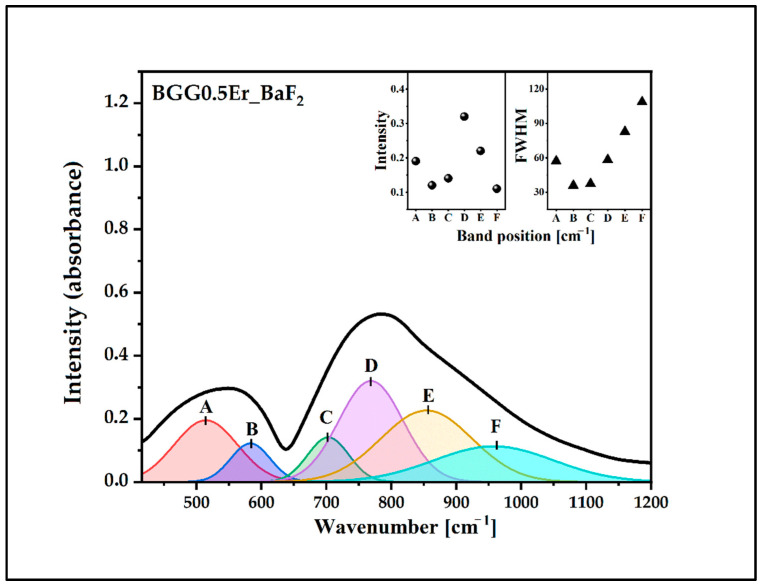
Deconvoluted IR spectrum of BGG0.5Er_BaF_2_ glass.

**Figure 7:**
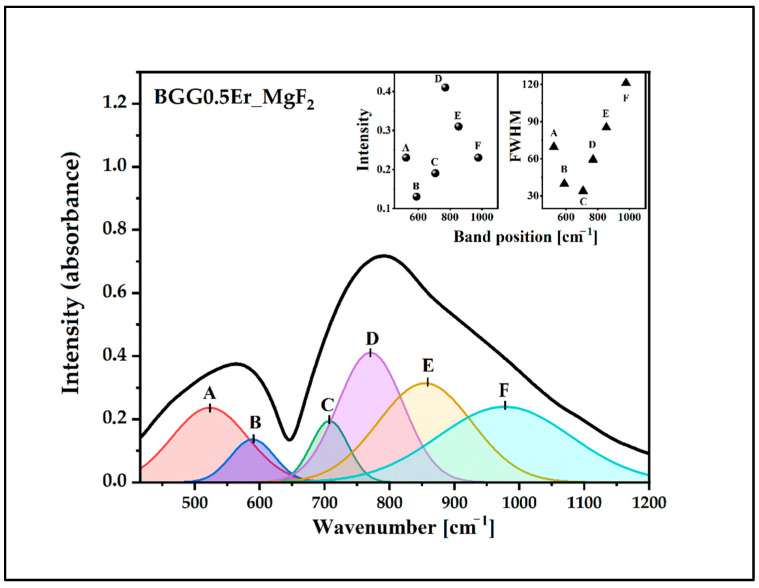
Deconvoluted IR spectrum of BGG0.5Er_MgF_2_ glass.

The IR spectra of the glasses were deconvoluted to investigate the changes in the BGG0.5Er glass network due to the addition of the fluoride compounds, as shown in Figure 4, Figure 5, Figure 6 and Figure 7. The assignments of the components bands to the vibrations are presented in Table 2. The six-component bands were present in each spectrum. At a lower wavenumber, the two-component bands (A and B in 490 cm^−1^–600 cm^−1^ range) can be attributed to bending vibrations involving X−O−X bridges (X used for Ge/Ga in tetrahedral coordination). The high-frequency region of 650 cm^−1^–950 cm^−1^ is composed of four bands: C, D, E, and F (Figure 4, Figure 5, Figure 6 and Figure 7). The band C at 700 cm^−1^–730 cm^−1^ can be attributed to the symmetrical stretching vibration of the ^[6]^Ge-O-^[6]^Ge bonds from the GeO_6_ units. The band D (at 760 cm^−1^–800 cm^−1^) can be assigned to the asymmetrical stretching vibration of the ^[4]^Ge-O-^[4]^Ge bonds connecting the GeO_4_ units. The band E (at 850 cm^−1^–880 cm^−1^) can be attributed to the asymmetrical stretching vibration of the bridging oxygens (BO) from the GeO_4_ tetrahedra (^[4]^Ge-O-^[4]^Ge). The last band, F, at 950 cm^−1^–1000 cm^−1^) can be related to the stretching vibration of the non-bridging oxygens (NBO) of the GeO_4_ tetrahedra [32,33,34,35,36].

**Figure 8:**
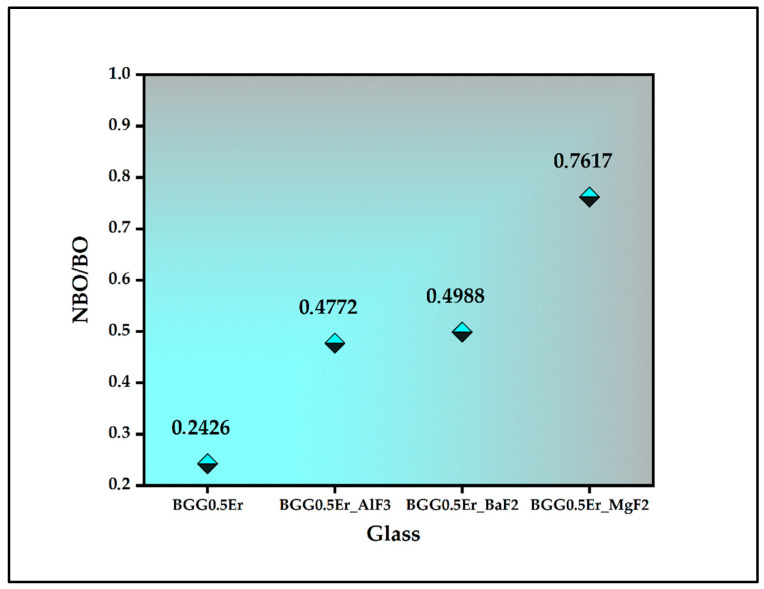
The ratio of the NBO/BO.

The parameters of the component bans are presented in the inset of Figure 4, Figure 5, Figure 6 and Figure 7 (the intensity and full width at half maximum (FWHM)). The assessment of the bands in the IR spectra indicates that the intensity of the bands from the BO and NBO vibrations change when the fluorides are added to the matrix. These promote the formation of NBO to the detriment of the bridging oxygens (BO) in the GeO_4_ tetrahedrons. As a result, the degree of polymerization of the BGG0.5Er glass network decreased while its NBO/BO ratio was 0.24. However, the NBO/BO ratio of the samples with cation size increases is as follows: < MgF_2_ (0.76)> BaF_2_ (0.49)> AlF_3_ (0.47) (Figure 8), which is in concordance with the results obtained by Guérineau et al., in which the addition of the Y_2_O_3_ and La_2_O_3_ in a BGG system caused the appearance of more NBO in the germanium and gallate polyhedra [5].

#### 3.3.2. Raman Spectra

**Figure 9:**
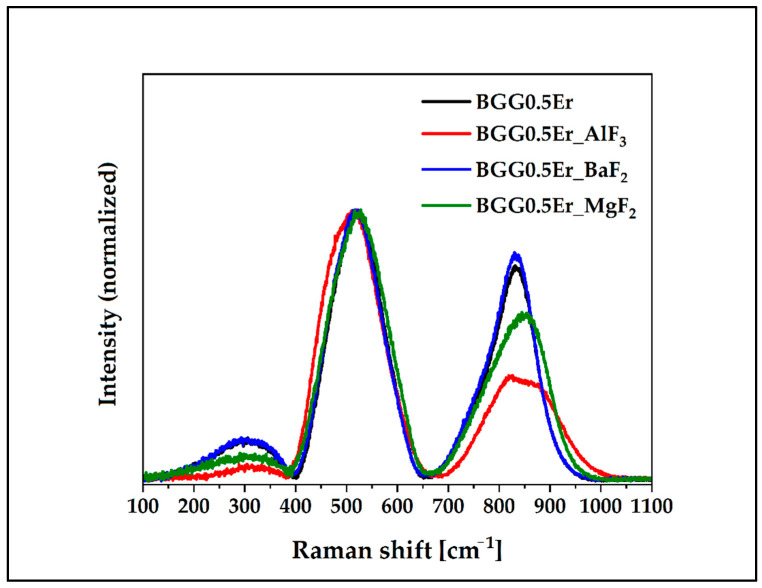
Normalized Raman spectra of glasses in the 100 cm^−1^–1100 cm^−1^ range.

The structural compositions of all composites were completed using Raman spectroscopy. Figure 9 presents the normalized Raman spectra for glasses in the 100–1100 cm^−1^. The three Raman signatures in the maximum at 300 cm^−1^, 500 cm^−1^, and 850 cm^−1^ are presented in Figure 9. Moreover, the two bands in the 200 cm^−1^–400 cm^−1^ and 650 cm^−1^–1100 cm^−1^ ranges have various intensities. Furthermore, the FWHM of the bands have different values, indicating that the modification of the structure occurred when the fluoride compounds were added.

**Figure 10:**
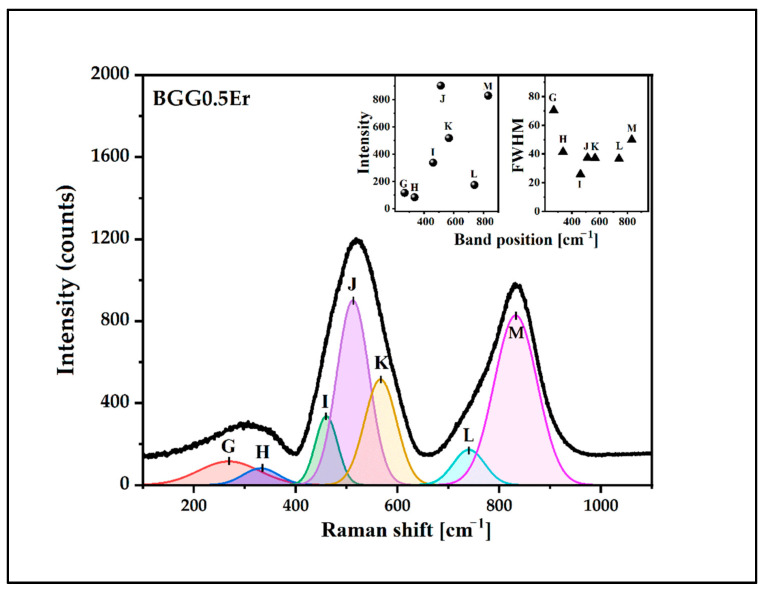
Deconvoluted Raman spectrum of BGG0.5Er glass.

**Figure 11:**
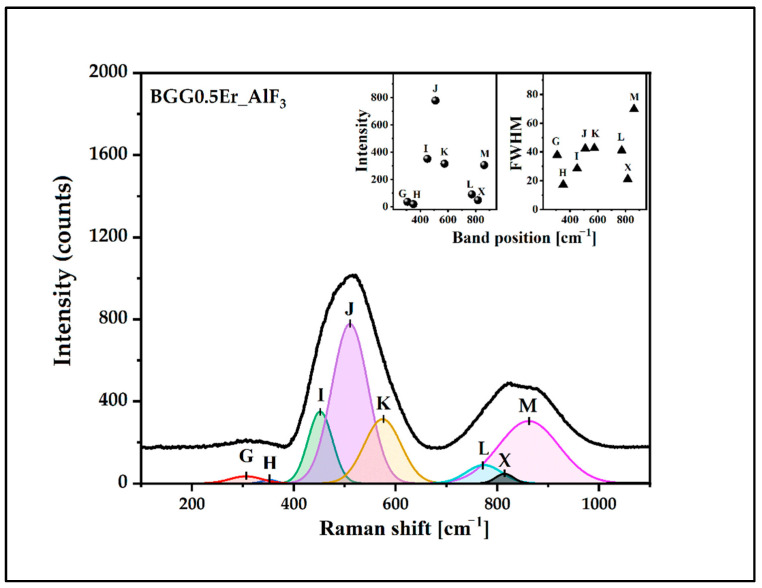
Deconvoluted Raman spectrum of BGG0.5Er_AlF_3_ glass.

**Figure 12:**
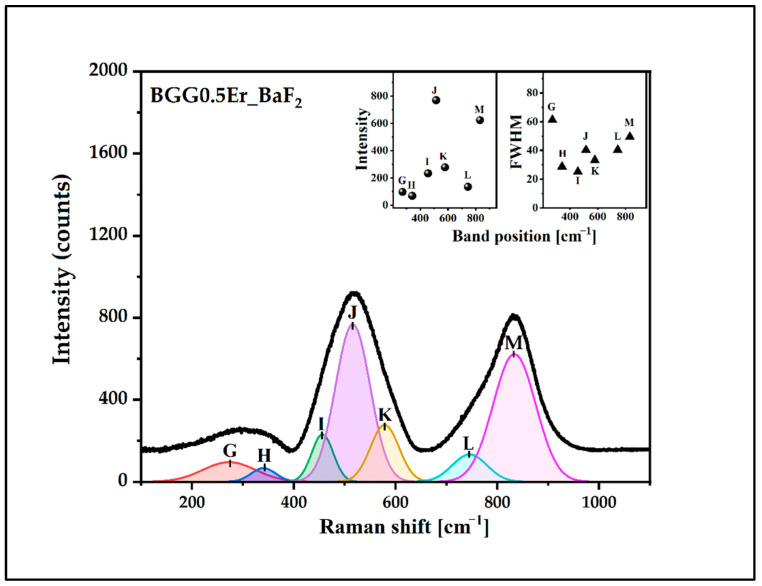
Deconvoluted Raman spectrum of BGG0.5Er_BaF_2_ glass.

**Figure 13:**
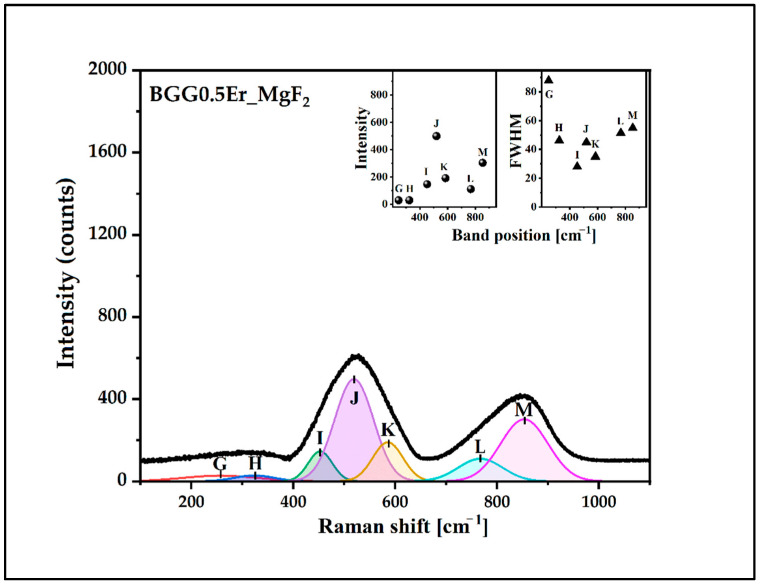
Deconvoluted Raman spectrum of BGG0.5Er_MgF_2_ glass.

The deconvoluted Raman spectra of the glasses were presented in Figure 10, Figure 11, Figure 12 and Figure 13. The assignment of the vibrations observed in the components bands (seven) is presented in Table 3. The component bands (G–M) are presented in each decomposed Raman spectrum. Additionally, the band X appeared in the decomposed Raman spectrum of the BGG0.5Er_AlF_3_ glass. The band G (at around 250 cm^−1^–300 cm^−1^) can be assigned to the bending vibration of the Ge-O-Ge bonds of the Ge(2) units. The band H at 320 cm^−1^–350 cm^−1^ can be attributed to the Ge,Ga-O-Ba vibration. The band I (at 450 cm^−1^–460 cm^−1^) is related to the symmetrical stretching vibration of the Ge,Ga-O-Ge,Ga bonds with a four-membered GeO_4_/GaO_4_ ring. The band J (at 500 cm^−1^ to 520 cm^−1^) is assigned to the vibration of BO (GeO_4_/GaO_4_) in three-membered rings. The band K (at around 560 cm^−1^–580 cm^−1^) can be attributed to the symmetrical stretching vibration of the GeO_6_ octahedral. The next band, L, (at 740 cm^−1^ to 770 cm^−1^) is related to the symmetrical stretching vibration of the NBO (Ge-O-) of Ge(2) units. The band X at around 812 cm^−2^ in the spectrum of the BGG0.5Er_AlF_3_ glass can be assigned to the symmetric stretching vibration of the Al-O-Al in the Al(4) units. The last band in each decomposed spectrum of glasses (M, at 830 cm^−1^–860 cm^−1^) corresponds to the symmetric stretching vibration of the NBO (Ge-O-) of the Ge(3) units [34,37,38,39,40,41,42]. Based on the Raman spectra studies, we assume that the addition of the fluoride compounds to the BGG_0.5Er glass results in decreases in the Ge(3) units at the expense of an increase in the Ge(2) units.

#### 3.3.3. ^27^Al MAS NMR Spectrum Analysis

**Figure 14:**
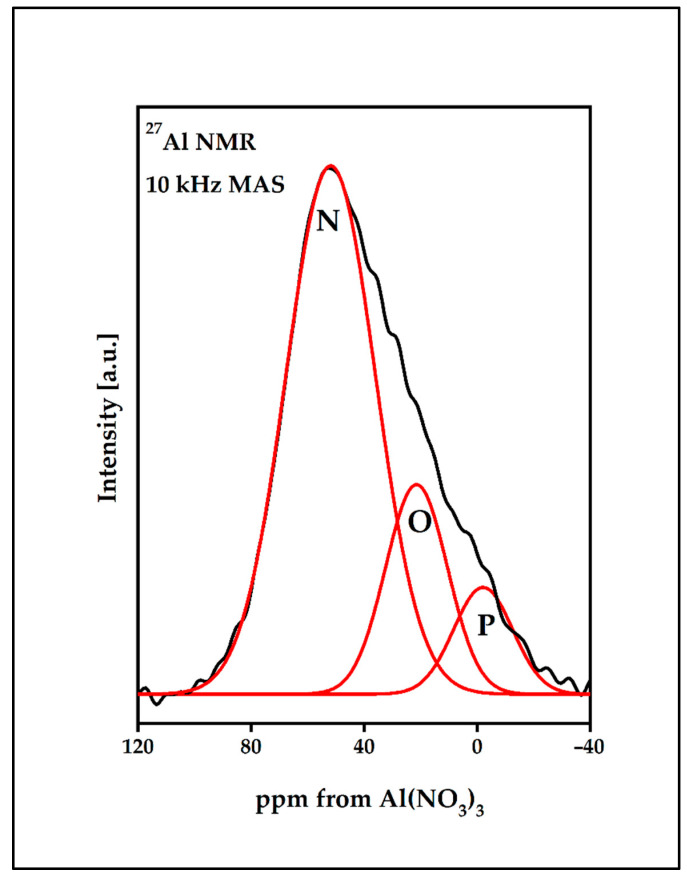
^27^Al MAS-NMR spectrum of BGG0.5Er_AlF_3_ glass.

Figure 14 presents the ^27^Al MAS-NMR spectrum of the BGG0.5Er_AlF_3_ glass. The analyzed spectrum shows a primary resonance with a maximum in the −3 ppm (band N), 20 ppm (band O), and 50 ppm (band P), which is characteristic of a six-, five-, and four-coordinated aluminum, respectively [43]. The parameters of the components band are given in Table 4.

### 3.4. Luminescence Properties

**Figure 15:**
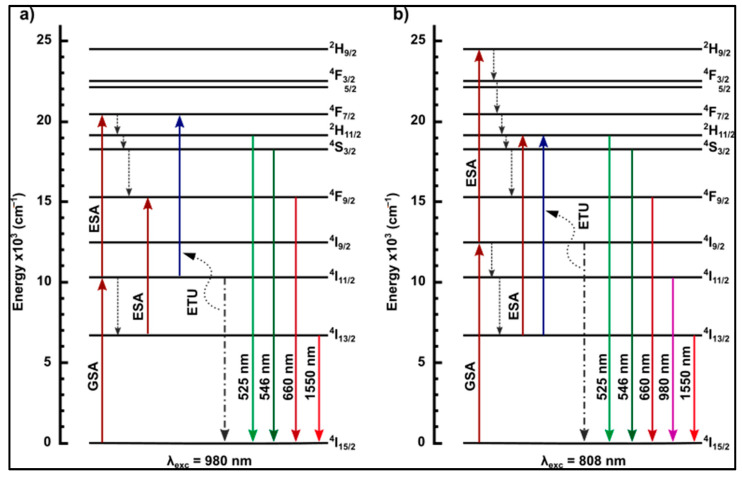
Energy level diagrams of Er^3+^-doped gallo-germanate glasses under laser excitation at (**a**) 980 nm and (**b**) 808 nm. GSA, ESA, non-radiative, and possible luminescence transitions have been indicated.

The luminescence measurements were performed by pumping the glasses with the two characteristic erbium absorption bands (980 nm and 808 nm) using a high-power semiconductor laser diode. The first one corresponds to the ^4^I_15/2_ → ^4^I_11/2_ energy transition (the ground state absorption phenomenon) and is widely used for the excitation of rare earth ions doped with glasses or optical fibers due to the high absorption cross-section (Figure 15a). The second absorption band is characterized by a lower absorption cross-section parameter, which corresponds to the ^4^I_15/2_ → ^4^I_9/2_ transition from GSA (Figure 15b). In this work, we analyze the energy flow in the context of two transition channels—Ch_980_ (^4^I_15/2_ → ^4^I_11/2_) and Ch_808_ (^4^I_15/2_ → ^4^I_9/2_). After GSA, the erbium ions in the Ch_808_ could non-radiatively relax to the ^4^I_11/2’s_ lower energy level, and therefore, another non-radiative relaxation to the ^4^I_13/2_ state can occur in both channels. Finally, the radiative transition ^4^I_13/2_ → ^4^I_15/2_ occurs, resulting in a strong luminescence band at 1.55 µm. Simultaneously, the erbium ions could absorb additional energy after the GSA process, thereby inducing a transition to higher energy states. Excited state absorption (ESA) and energy Transfer up-conversion (ETU) occur in both channels and result in ^4^I_11/2_ → ^4^F_7/2_, (Ch_980_), ^4^I_9/2_ → ^2^H_9/2_ (Ch_808_), and ^4^I_13/2_ → ^2^H_11/2_ (Ch_808_) transitions [44]. Afterward, the non-radiative relaxation processes can cause erbium ions to move to lower energy levels where it becomes possible to observe the radiative transitions in both channels: ^2^H_11/2_ → ^4^I_15/2_ (525 nm), ^4^S_3/2_ → ^4^I_15/2_ (546 nm), and ^4^F_9/2_ → ^4^I_15/2_ (660 nm). The luminescence spectra observed in the near-infrared and visible range are the result of the energy flow of the erbium ions in down- and up-conversion processes. Since both phenomena are mutually competitive, it was possible to observe changes in the intensity of the luminescence in particular bands during modification of the composition of the matrix [29,45]. Further analysis will allow for the determination of energy flow channels as a function of different fluoride compounds in the glass matrix.

#### 3.4.1. Luminescence Results under the 980 nm Excitation Pump

**Figure 16:**
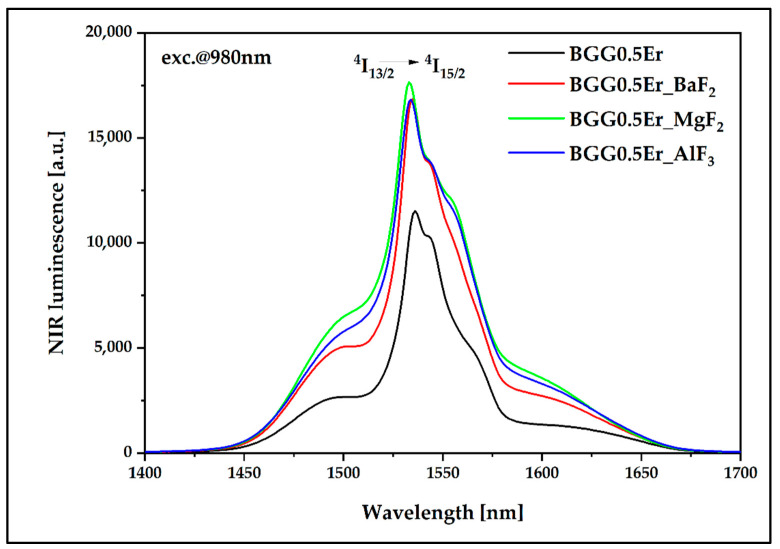
Luminescence spectra of Er^3+^-doped BGG glass modified by different fluoride compounds under 980 nm laser excitation.

**Figure 17:**
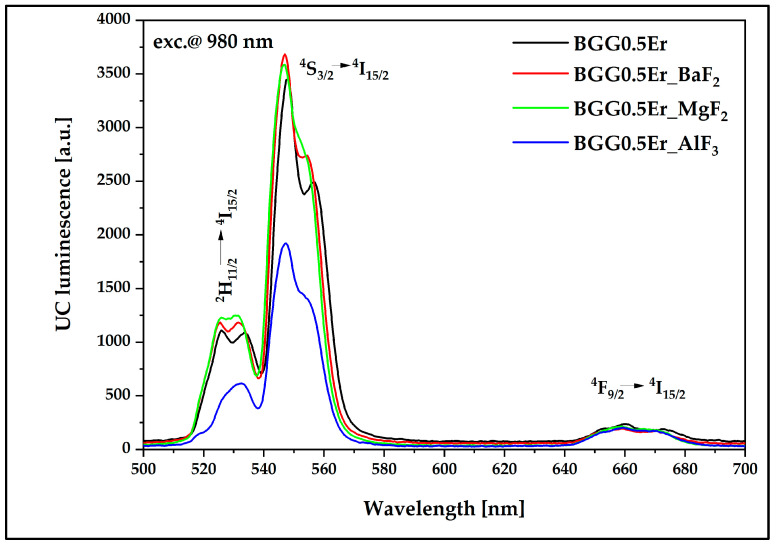
UC luminescence of Er^3+^-doped BGG glass modified by different fluoride compounds under 980 nm laser excitation.

Figure 16 and Figure 17 present the luminescence effect of using fluoride modifiers (BaF_2_, MgF_2_, AlF_3_) in the matrix. It is possible to observe that the samples with the fluoride components showed an enhancement of the emission intensity in the near-infrared range located at 1400–1700 nm (Figure 16), which is related to the radiative energy transition ^4^I_13/2_ → ^4^I_15/2_. Regarding the visible range from 500 to 700 nm (Figure 17), three luminescence bands at 525 nm (^2^H_11/2_ → ^4^I_15/2_), 546 nm (^4^S_3/2_ → ^4^I_15/2_), and 660 nm (^4^F_9/2_ → ^4^I_15/2_) were identified. All bands of the visible range exhibited a slight increase in the luminescence intensity, except for the glassy matrix containing MgF_2_, for which the decrease was significant at 546 nm and 660 nm. To explain this phenomenon, it is necessary to analyze the possible energy transitions inside the erbium ions during pumping radiation using a 980 nm light source (Figure 15a). After the ground state absorption, excited state absorption, and energy transfer up-conversion processes, erbium ions from the ^4^F_7/2_ can non-radiatively transit to the ^2^H_11/2_ excited energy level, from where a radiative transition to the ^4^I_15/2_ ground level can take place, resulting in the emission of an electromagnetic wave with a wavelength at 525 nm. On the other hand, excited erbium ions can transit non-radiatively from ^2^H_11/2_ to the ^4^S_3/2_ and ^4^F_9/2_ lower energy levels; from here, there can be radiative transitions to the ground energy level, which are responsible for generating the two visible radiative bands at 546 nm and 660 nm, respectively [24,46].

The only variable in this work is the introduction of fluorides as glass-forming components. Barium and magnesium fluorides have practically no effect on the energy flow channels in the visible range compared to the BGG0.5Er, while the addition of aluminum fluoride reduces the energy flow channel from ^4^F_7/2_ to lower energy levels. One bridging oxygen anion in the glass network is replaced by two non-bridging fluorine anions when fluorine is added to the oxide glass. It is well known that an increasing number of non-bridging oxides (NBO) in a glass structure leads to a decrease in phonon energy of the host glass and promotes the up-conversion mechanisms [47]. However, due to the smallest ionic radius (0.51 Å) and the highest field strength (11.53 Å^−2^) of Al^3+^ among the other analyzed fluorides, the aluminum ions modify, similarly to glass-forming ions in the gallo-germanate glass network. The absorbance band (labeled as X) is observed in the Raman spectrum of the BGG0.5Er_AlF_3_ glass (Figure 11). The presence of this band indicates that the chemical bonds are stronger in the glass network and the maximum phonon energy is slightly shifted towards a higher value. Aluminum-glass network atoms reduce the probability of ^2^H_11/2_ → ^4^I_15/2_ and ^4^S_3/2_ → ^4^I_15/2_ radiative transitions and promote a non-radiative energy transition [28,47].

**Figure 18:**
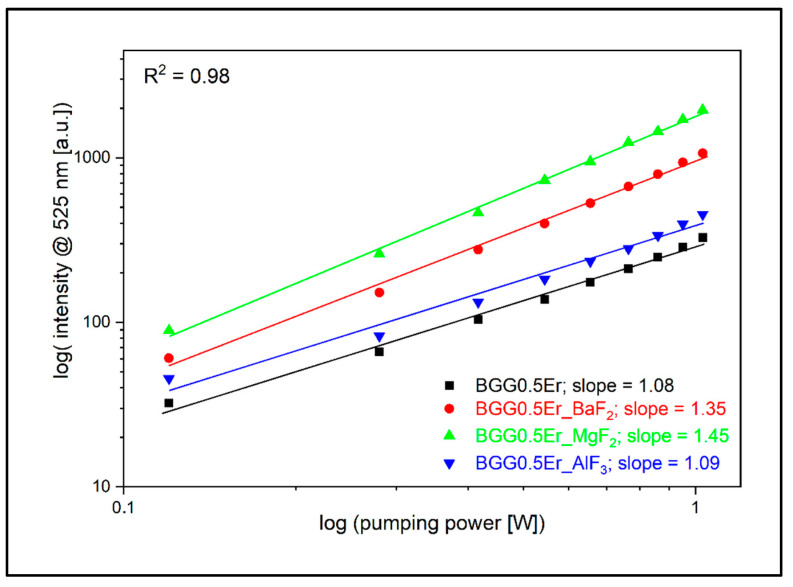
Log–log plot of up-conversion emission at 525 nm of fabricated glasses.

**Figure 19:**
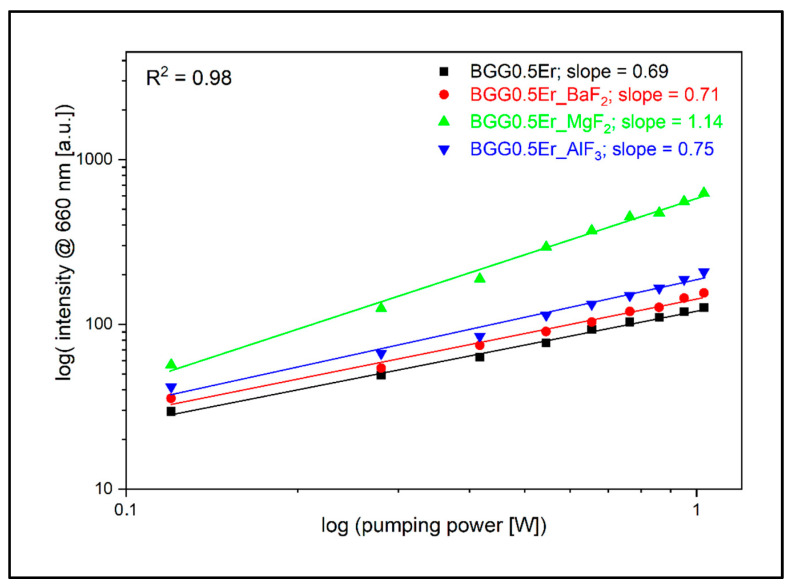
Log–log plot of up-conversion emission at 546 nm of fabricated glasses.

To take a closer look at the process of energy conversion from the IR range to the visible range, we analyzed the luminescence signal in the 525 nm (Er^3+^: ^2^H_11/2_ → ^4^I_15/2_), 546 nm (Er^3+^: ^4^S_3/2_ → ^4^I_15/2_), and 660 nm (Er^3+^: ^4^F_9/2_ → ^4^I_15/2_) emission bands as a function of the pump radiation intensity (λ_exc_ = 980 nm). The obtained energy slope values indirectly allow for the determination of the E_IR_ → E_VIS_ conversion efficiency. Considering the 525 nm band, the BGG0.5Er glass can be characterized by the smallest value of the slope, equal to 1.08, while the highest value of 1.45 was determined for the BGG0.5Er_MgF_2_ glass (Figure 18). In a situation where there are no unfavorable depopulation processes (non-radiative transitions—multiphonon; energy migration; luminescence quenching), the conversion values are equal to 2.0 due to the emission occurring via two-photon absorption—GSA and ESA [14,48]. A similar correlation is observed in the 546 nm luminescence band; the slope values are in the range from 1.27 in the BGG0.5Er sample to 1.46 in the BGG0.5Er_MgF_2_ glass (Figure 19).

**Figure 20:**
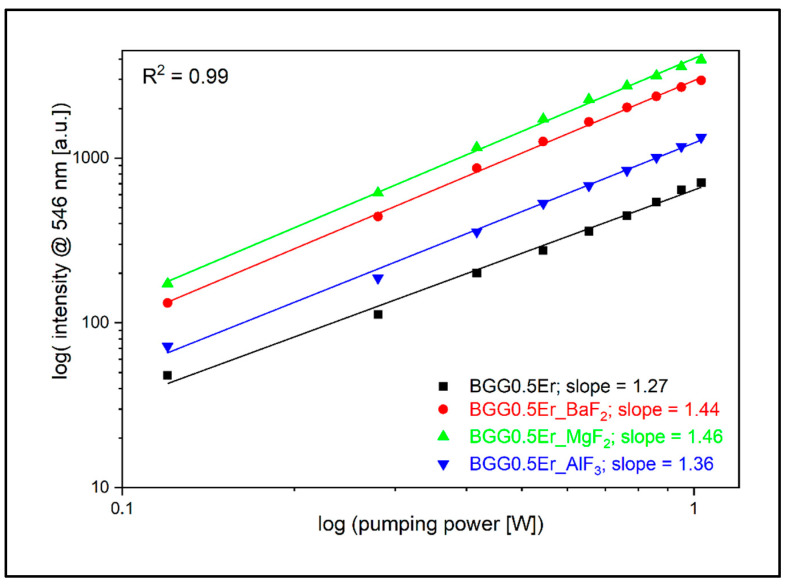
Log–log plot of up-conversion emission at 660 nm of fabricated glasses.

Significantly smaller values of the slope parameter were observed during the analysis of the pump radiation power’s impact on the emission intensity at the wavelength of 660 nm. Although, as in the investigation of the other bands in the visible range, the minimum value of the slope is characterized by the sample BGG0.5Er, which equals 0.69, and the largest value of 1.14 is characterized by the sample BGG0.5Er_MgF_2_. Erbium ions after GSA and a non-radiative Er^3+^: ^4^I_11/2_ → ^4^I_13/2_ transition relax to the ^4^I_15/2_ ground level and emit radiation at a wavelength of 1.55 μm. Simultaneously, the excited state absorption Er^3+^: ^4^I_11/2_ → ^4^F_9/2_ can occur (Figure 15a). Due to the competitive nature of these phenomena, the rate of the ^4^F_9/2_ (due to the ESA process) level population is limited by the effective Er^3+^: ^4^I_13/2_ → ^4^I_15/2_ radiative transition, as evidenced by the luminescence research and, further, by the obtained low values of the slope parameter for the 660 nm emission band (Figure 20).

The obtained slope parameters in our research prove the existence of harmful multiphonon depopulation processes. Glass modified with MgF_2_ is characterized by the highest luminescence for both the IR and visible range among all of the examined samples. Erbium incorporated into the immediate surroundings of magnesium ions causes an increase in the ratio of the probability of the occurrence of radiative transitions in comparison to non-radiative transitions, which is also particularly evident from the obtained slope parameter values. The predominant non-radiative transitions in other matrices result in slope values at levels below 1.0. The addition of fluorides as glass-forming components and further incorporation of F^−^ anions into the glass reduce this feature as the maximum phonon energy decreases in comparison to the BGG0.5Er, a glass based only on oxides [46,47]. The highest energy slope value has been observed in glass with the magnesium fluoride component due to the influence of Mg^2+^ cations on the depolymerization of the BGG0.5Er glass structure.

#### 3.4.2. Luminescence Results under the 808 nm Excitation Pump

**Figure 21:**
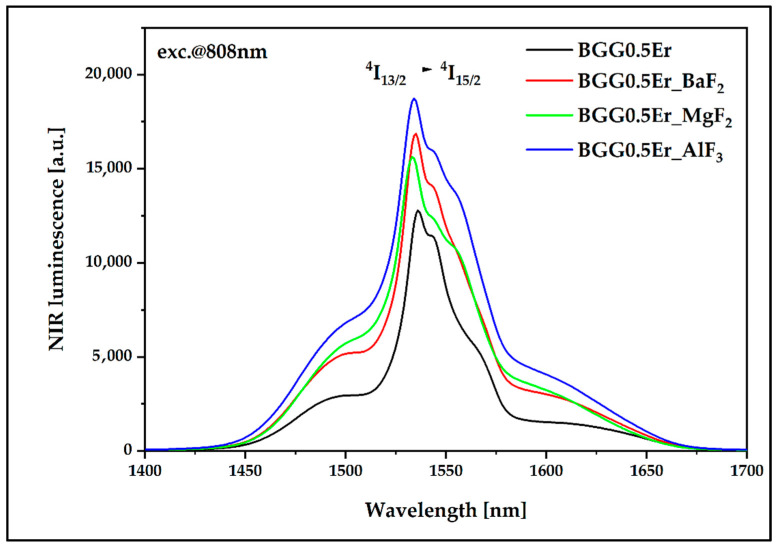
Luminescence spectra of Er^3+^-doped BGG glass modified by different fluoride compounds under 808 nm laser excitation in the 1400–1700 range.

During further research, the synthesized glass matrices were investigated in terms of the luminescent properties under excitation radiation at 808 nm. Regarding the emission in the near-IR range (Figure 21), the profile of the luminescence spectrum is identical to that observed when pumping at 980 nm. At the same time, it is visible that, in the samples with the fluoride glass-forming components, the probability of the energy flow channel related to the occurring ^4^I_13/2_ → ^4^I_15/2_ radiative transition is higher than in the glass matrix based solely on oxides (BGG0.5Er).

**Figure 22:**
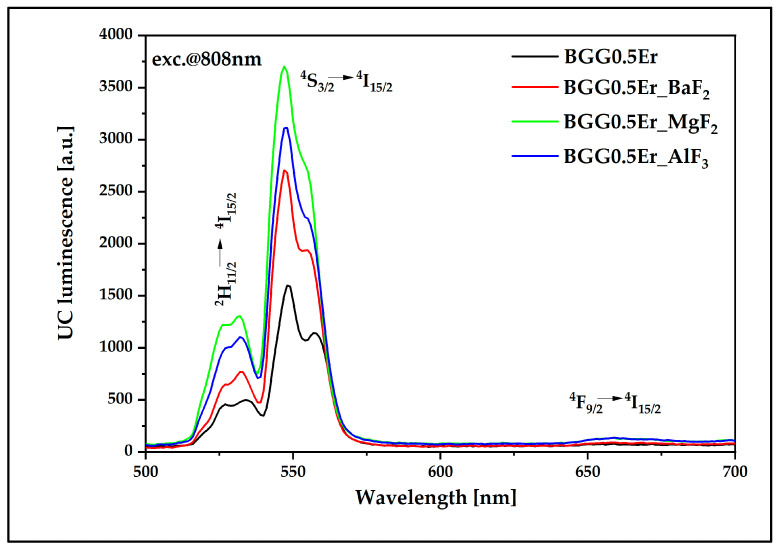
UC luminescence of Er^3+^-doped BGG glass modified by different fluoride compounds under 808 nm laser excitation.

In the visible spectrum range, we observed a significant decrease in the signal at 660 nm, which is related to the radiative transition ^4^F_9/2_ → ^4^I_15/2_ (Figure 22). After the GSA and ESA processes, the probability of emitting energy from the upper levels (^2^H_11/2_ and ^4^S_3/2_) to the radiative transitions (^4^I_15/2_—ground level) is higher than the probability of a non-radiative transition (^4^F_9/2_ lower energy level), as observed in Figure 15b. The presence of fluorides in the glass matrix leads to an almost complete blockage of the non-radiative relaxation channel, simultaneously promoting the channels associated with luminescent transitions [24,46]. Glass with the addition of MgF_2_ is characterized by the highest luminescence intensity in the emission bands centered at the wavelengths of 525 nm and 546 nm, while the base–oxygen glass BGG0.5Er has a smaller probability for luminescence transitions in the visible range.

**Figure 23:**
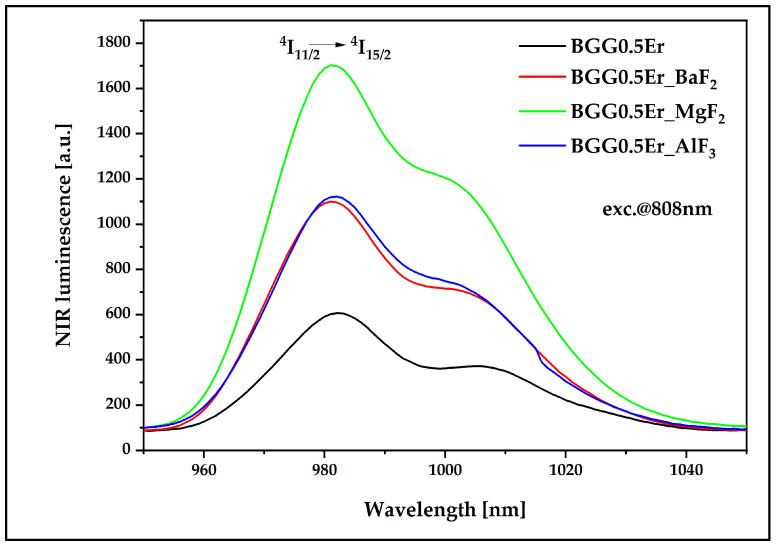
Near-infrared luminescence spectra of Er^3+^-doped BGG glass modified by different fluoride compounds under 808 nm laser excitation.

To fully analyze the energy flow channels, the near-infrared luminescence research was performed in the band at 980 nm, which is related to the radiative transition Er^3+^: ^4^I_11/2_ → ^4^I_15/2_. The results presented in Figure 23 indicate the opening of this emission channel, which is associated with the addition of fluorides into the glass matrix. In particular, the influence of MgF_2_ on the efficiency of the energy conversion *n* in this band is significantly positive since it shows the highest emission intensity among all of the synthesized samples.

## 4. Discussion

AlF_3_, BaF_2_, and MgF_2_ were substituted for BaO in the BaO-Ga_2_O_3_-GeO_2_ glass doped with erbium ions to change the local structure of the gallo-germanate glass (BGG). According to the obtained results, by adding AlF_3_, BaF_2_, and MgF_2_ into the glass host, the luminescence properties of erbium ions are enhanced. Based on infrared and Raman spectroscopies, it has been proven that the presence of AlF_3_, BaF_2_, and MgF_2_ in the BGG glass host causes an increase in the number of non-bridging oxygens due to the depolymerization of the gallo-germanate glass network. It is well-known that the field strength of cations strongly influences the structure of glasses and their suitability for various applications. The changes in the BGG glass structure can be explained as relating to the effects of network-modifying cation field strength (cation field strength = Z/r^2^, where Z = cation charge, r = cation radius in Å) [49]. Based on the structural spectra study of the glasses, it can be concluded that barium and magnesium prefer the role of network modifiers. In the case of the AlF_3_, the aluminum acts as a glass network (in the coordination of four), as well as a network modifier (coordinations of five and six) in the network of the BGG0.5Er glass. In each of the glass networks, the presence of the Ge(2), and Ge(3) units was confirmed. Due to magnesium’s field strength, which is higher than the rest of the cations, Mg^2+^ prefers to associate with more Ge(2) units than aluminum and barium. According to a crystal-chemistry approach based on a modified random network model, a depolymerized glass network increases the regularity of sites occupied by lanthanide ions [11]. The luminescence studies of erbium-doped glasses modified with fluoride compounds confirmed that the addition of fluorides leads to an enhancement in the NIR emission. The most intensive UC luminescence was observed in the sample with MgF_2_ in both excitation schemes (808 and 980 nm).

## 5. Conclusions

In this paper, a detailed analysis of the structural and luminescence properties of erbium-doped gallo-germanate glass modified by fluoride compounds (BaF_2_, MgF_2_, and AlF_3_) was performed. Based on the deconvoluted IR and Raman spectra, it was corroborated that the addition of the fluoride compounds into the BGG_0.5Er glass resulted in a decrease in its glass network polymerization. The decrease in the number of Ge(3) units at the expense of an increase in the Ge(2) units was found in the decomposed Raman spectra of each glass. The highest NBO/BO ratio was obtained for the BGG glass with the MgF_2_. In the glass network of the aluminum-modified BGG glass doped with Er^3+,^ the presence of Al with different coordinations (tetra-, penta- and octahedral) was confirmed based on the ^27^Al MAS NMR spectrum. For each glass, the most robust depolymerized network enhanced the regularity of the sites occupied by the erbium ions. The luminescence properties of the erbium ions in glasses were related to the results of the structural studies. In the case of visible emission, the green emission corresponding to the ^2^H_11/2_ and ^4^S_3/2_ levels was dominated in both of the used excitation channels. It is worth noticing that the luminescence profile depends on the fluoride modifiers employed and the excitation wavelength. Our experiment gives valuable information about luminescence behavior in relation to the structural modification of the host. In our further investigation, the next step will be the fabrication of transparent glass ceramics in the BGG0.5Er_MgF_2_ system. The BGG glass doped with erbium and modified by MgF_2_ has better emission intensities and the lowest thermal stability of the other glasses.

## Figures and Tables

**Table 1:** Thermal properties of erbium-doped BGG glass modified by fluorides.

Glass	T_g_ [°C]	T_x1_ [°C]	T_x2_ [°C]	T_x3_ [°C]	ΔT [°C]
BGG0.5Er	630	836	-	-	206
BGG0.5Er_BaF_2_	595	735	792	-	140
BGG0.5Er_MgF_2_	585	642	871	-	57
BGG0.5Er_AlF_3_	610	735	754	1030	125

**Table 2:** Assignment of the IR bands.

Bands	Assignment
A	bending vibrations of X−O−X bridges (X used for Ge/Ga in tetrahedral coordination)
B	
C	symmetrical stretching vibration of the ^[6]^Ge-O-^[6]^Ge bonds from GeO_6_ units
D	asymmetrical stretching vibration of the ^[4]^Ge-O-^[4]^Ge bonds connecting GeO_4_ units
E	asymmetrical stretching vibration of the bridging oxygens (BO) from GeO_4_ tetrahedra
F	stretching vibration of the non-bridging oxygens (NBO) of GeO_4_ tetrahedra

**Table 3:** Assignment of the bands in Raman spectra of glasses.

Bands	Assignment
G	bending vibration of the Ge-O-Ge bonds of Ge(2) units
H	Ge,Ga-O-Ba vibration
I	symmetrical stretching vibration of the Ge,Ga-O-Ge,Ga bonds with a 4-membered GeO_4_/GaO_4_ ring
J	the vibration of BO (GeO_4_/GaO_4_) in 3-membered rings
K	symmetrical stretching vibration of the GeO_6_ octahedral
L	symmetrical stretching vibration of the NBO (Ge-O-) of Ge(2) units
M	symmetrical stretching vibration of the NBO (Ge-O-) of Ge(3) units
X	symmetric stretching vibration of the Al-O-Al in Al(4) units

**Table 4:** Deconvolution of ^27^Al MAS-NMR spectrum.

Glass	Peak Position [ppm]	FWHM [ppm]	Relative Intensity [%]
BGG0.5Er_AlF_3_	52.6	37.1	71
	22.2	25.7	20
	−1.3	24.5	9

## Data Availability

Not applicable.
